# The impact of an audience response system on a summative assessment, a controlled field study

**DOI:** 10.1186/s12909-020-02130-4

**Published:** 2020-07-13

**Authors:** Thorsten Schmidt, Anastasia Gazou, Angelika Rieß, Olaf Rieß, Kathrin Grundmann-Hauser, Ruth Falb, Malou Schadeck, Tilman Heinrich, Mahkameh Abeditashi, Jana Schmidt, Ulrike A. Mau-Holzmann, Kai P. Schnabel

**Affiliations:** 1 Institute of Medical Genetics and Applied Genomics, University of Tuebingen, Calwerstrasse 7, 72076 Tuebingen, Germany; 2 Institute for Medical Education, Department for Education and Media, University of Bern, Bern, Switzerland

**Keywords:** Audience response system, Classroom response system, eduVote, Assessment, Feedback

## Abstract

**Background:**

Audience response systems allow to activate the audience and to receive a direct feedback of participants during lectures. Modern systems do not require any proprietary hardware anymore. Students can directly respond on their smartphone. Several studies reported about a high level of satisfaction of students when audience response systems are used, however their impact on learning success is still unclear.

**Methods:**

In order to evaluate the impact of an audience response system on the learning success we implemented the audience response system eduVote into a seminar series and performed a controlled crossover study on its impact on assessments. One hundred fifty-four students in nine groups were taught the same content. In four groups, eduVote was integrated for the first topic while five groups were taught this topic without the audience response systems. For a second topic, the groups were switched: Those groups who were taught before using eduVote were now taught without the audience response system and vice versa. We then analysed the impact of the audience response system on the students’ performance in a summative assessment and specifically focused on questions dealing with the topic, for which the audience response system was used during teaching. We further assessed the students’ perception on the use of eduVote using questionnaires.

**Results:**

In our controlled crossover study we could not confirm an impact of the audience response system eduVote on long-term persistence i.e. the students’ performance in the summative assessment. Our evaluation revealed that students assessed the use of eduVote very positively, felt stronger engaged and better motivated to deal with the respective topics and would prefer their integration into additional courses as well. In particular we identified that students who feel uncomfortable with answering questions in front of others profit from the use of an audience response system during teaching.

**Conclusions:**

Audience response systems motivate and activate students and increase their engagement during classes. However, their impact on long-term persistence and summative assessments may be limited. Audience response systems, however, specifically allow activating students which cannot be reached by the traditional way of asking questions without such an anonymous tool.

## Background

*Audience response* systems, also known as *classroom response* systems, are tools to activate learners during a lecture, to motivate them to participate and to actively follow the instruction. Moreover, tutors can use audience response systems to monitor the learning progress, to receive a fast feedback and to flexibly adapt the contents of the lecture to the learners’ needs.

Today, most students (95% in the age group of 14–29 years) [1] own a smartphone. Therefore, proprietary audience response systems, requiring high efforts as well as high purchase and running costs are no longer required as smartphone-base audience response systems can be used. Browser-based audience response systems do not even require the installation of an app. Such systems are e.g. SMILE (Smartphones in der Lehre) [2, 3], eduVote (SimpleSoft - Buchholz Wengst GbR, Braunschweig, Germany) [4] or Socrative (Showbie Inc., Edmonton, AB). They are versatile and just require a device with internet connection and a web browser. While SMILE can be used for free, both eduVote and Socrative required a paid license. In comparison with traditional systems, Socrative was found to support an active participation in the lecture [5].

The use of audience response systems is highly accepted among students. Students positively assess their use [5–7] and audience response systems were reported to increase the student’s satisfaction with a certain lecture.

There are contradictory results regarding the impact of audience response systems on the results of assessments [6–9]. However, many of such studies were done in lectures and using proprietary audience response systems. There are less or even no studies reported about the use of audience response systems in seminars and regarding smartphone-based systems like e.g. eduVote.

For this reason, we integrated the smartphone-based audience response system eduVote into our classes. EduVote allows a direct integration into PowerPoint presentations. Students access eduVote on their smartphone using a specific website. The students are then able to answer a specific question on their smartphone once it is activated by the lecturer. With the press of a button, the answers of the participants directly appear as bar charts in the PowerPoint presentation in a summarized fashion and can be discussed with the audience.

Here we tested the impact of the audience response system eduVote in a seminar setting on the results of a summative assessment as well as on the students’ perception of the use of this audience response system during class.

## Methods

### Study setting and design

This study was conducted in Summer term 2019 as crossover study [10] during the seminar series “Human genetics”. This seminar series is taught by the Institute of Medical Genetics and Applied Genomics at the University of Tuebingen for the study program in Human Medicine (for students in their 10th semester / 6th clinical semester). The seminar is part of the curricular teaching program in Human Genetics and complements a lecture series in “Applied Human Genetics - Clinical Genetics Part I” (5th semester / 1st clinical semester) and “Part II” (10th semester / 6th clinical semester). While the lectures were held in the plenum of all students, the 154 students of the seminar “Human Genetics” were taught the same contents for different topics in nine seminar groups (groups A-I). Collective 1 (seminar groups A-D) were asked interactive multiple-choice questions using the audience response system while they were taught topic 1. However, topic 2 was taught without this interactive part (i.e. the questions were included in the presentation and they were briefly discussed but students were not asked for their individual answers). Collective 2 (seminar groups E-I) received interactive questions during topic 2 and accordingly topic 1 was taught without this interactive part (see above).

In their summative assessment (took on average 3 weeks (23.9 days) after the last seminars included in this study and 1 week (8.5 days) after the end of the whole seminar series) all groups were asked questions both for topic 1 and topic 2. For analysis, we correlated the success rate in the summative assessment (i.e. the percentage of correct answers) for topic 1 and 2 with the affiliation to collective 1 and 2 (and thereby with the use of interactive questions for the specific topic), respectively.

The seminar tutors were highly experienced experts in the seminar topic. Most tutors conducted the seminar already many times for years and attended a dedicated training in didactics. During the whole seminar series, different teaching-learning methods, adapted to the topic of the respective seminar, were used including collective discussion, problem solving, the use of simulated patients, interactive drawings of family trees, instruction in a lecture-based format, questions to (re-)activate the students etc.

In anonymous questionnaires, we further assessed the students’ demographics, their usual behaviour if a tutor asks questions and their perception of the use of the audience response system. These questionnaires were completed by the students immediately after the respective seminar with the audience response system was taught. The questionnaire was self-developed with external expertise and evaluated in a pilot study [11].

### Sample definition and recruitment of participants

Each semester, about 154 students of Human Medicine participate in the seminar “Human Genetics”. All participants of the seminar were invited to participate in the study. The participation in the research study was voluntary and had no impact on the students' grades or whether they passed the course. No part of the study was linked to any form of incentives based on the answer given. The results of the final assessment only of those students who gave their written consent were included in the study. The students were further invited to complete an anonymous questionnaire. The sample consists of a) all students who gave their written consent to a statistical analysis of their summative assessment and of b) all students who completed an anonymous questionnaire, respectively.

### Statistical procedure

The dependent variable was the affiliation to either collective 1 (groups A-D) or collective 2 (groups E-I) and the measuring instrument the results of the summative assessment for topic 1 and 2. We used a paired t-test and compared the assessment results with the audience response system (collective 1: results for topic 1 + collective 2: results for topic 2) with the assessment results without the audience response system (collective 1: results for topic 2 + collective 2: results for topic 1).

### Sample size estimation

The sample size of about 154 students participating in this study was comparable to the number of participants in other studies which achieved statistically significant results [5–7]. Due to the use of dummy coding, each student was both part of the intervention and of the control cohort. This means that a student who e.g. attended for topic 1 a seminar with the audience response system and for topic 2 a seminar without the audience response system served both as proband for topic 1 and as control for topic 2. The statistical total number of cases was therefore 2*n = 308.

## Results

### Study setting

**Fig. 1:**
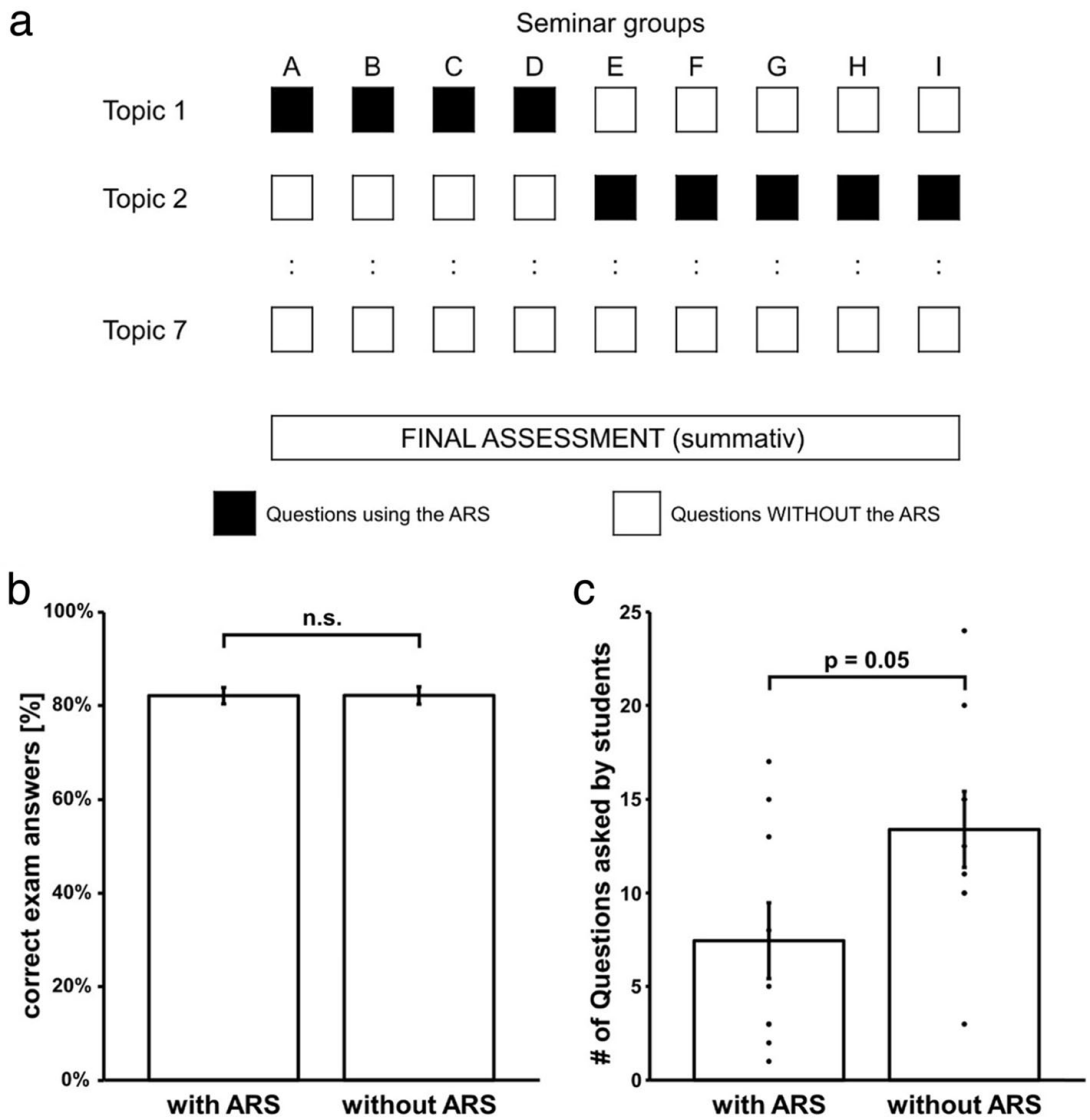
Study Design and results. **a** In the seminar series, 154 students of medicine in nine seminar groups were instructed in the same topic (A-I). When the students in seminar groups a-d were instructed in topic 1, they answered interactive questions using the audience response system (ARS), while topic 2 was taught without this interactive part. This design was flipped in seminar groups E-I. We analysed the impact of the audience response system on the results of the final summative assessment. **b** The results of the summative assessment were independent of the use of the audience response system (ARS). Shown is the mean % (± SEM) of correct answers given by students in the summative assessment for the respective topic for which the audience response system was used during teaching (with ARS, 82.1% ± 1.7%) and for the respective topic for which no audience response system was used (without ARS, 82.2% ± 1.9%). There was no difference between both groups (paired t-test, *p* = 0.98). **c** Questions of students in relation to the use of an audience response system. When no audience response system (ARS) was used the students tendentially (*p* = 0.055) asked more questions (13.4 ± 2.0) than in seminars in which the ARS was employed (7.4 ± 2.0). Mean ± SEM

In order to study the impact of an audience response system on a summative assessment in a seminar setting, we chose the seminar series “Human Genetics”. This seminar series consist of seven different seminars/topics and every student/group was required to attend all seven seminars/topics. Participation of the students was tracked. Either topic 1 (“autosomal recessive diseases”) or topic 2 (“autosomal dominant diseases”) was taught using the audience response system eduVote (SimpleSoft - Buchholz Wengst GbR, Braunschweig, Germany) (Fig. 1a).

### Participant demographics

One hundred fifty-four students participated in the nine groups of the seminar with a mean of 16 students (*n* = 9 to 22) per group. One hundred fourteen students (74% of 154) further completed an anonymous questionnaire. This allowed us to further specify the participant demographics: The study cohort consisted of 51% male and 48% female students (1% preferred not to specify the gender). 49% of students were in the age group of 21–25 years. One third (34%) was between 26 and 30 years old and 17% were older than 31 years (Table 1).

**Table 1:** Participant demographics. Listed is the number of study participants who completed an anonymous questionnaire (the percentage is listed in brackets). Deviations from the total number of participants are due to missing answers

Demographic	Participants (***n*** = 114)
Gender	
Male	57 (51%)
Female	54 (48%)
Diverse	0 (0%)
Prefer not to answer	1 (1%)
Age groups	
21–25	54 (49%)
26–30	37 (34%)
31–35	16 (15%)
> 35	2 (2%)

### Impact of the audience response system on the assessment results

One hundred nineteen students (77% of all seminar participants) gave their written consent that the results of their final assessment could be statistically analysed for this study. Sixty-one students (51% of 119) were taught in topic 1 (“autosomal recessive diseases”) with the use of the audience response system (ARS). For 58 students (49% of 119), the ARS was used for topic 2 (“autosomal dominant diseases”). During the 90 min of the seminar, approximately every 30 min (mean 28 min) the participants answered a multiple-choice question with five discriminators. The aggregated results of all students were displayed after the poll and all answers were subsequently discussed with the audience. We thereby provided a formative feedback to all students who participated in the poll. The respective other topic was taught without the use of eduVote; i.e. the respective questions were shown to the students but no individual answers were demanded and thereby no formative feedback was given. The summative assessment contained additional multiple-choice questions with five discriminators for either topic. These questions were different to the ones asked in the seminar but covered the respective topic (6 questions for topic 1 and 5 questions for topic 2).

We then analysed the answers given by each student in the summative assessment with regard to whether the audience response system (ARS) was employed in the seminar the student attended for the specific topic and calculated the percentage of correct answers with ARS and without ARS for each student. We then compared the percentages for all students but could not identify a difference between both cohorts (Fig. 1b). We concluded that the results of the summative assessment were independent of the use of the audience response system (ARS) in the respective seminar i.e. that the use of the ARS had no impact on the assessment results.

### Participant’s perception on the use of an audience response system

**Fig. 2:**
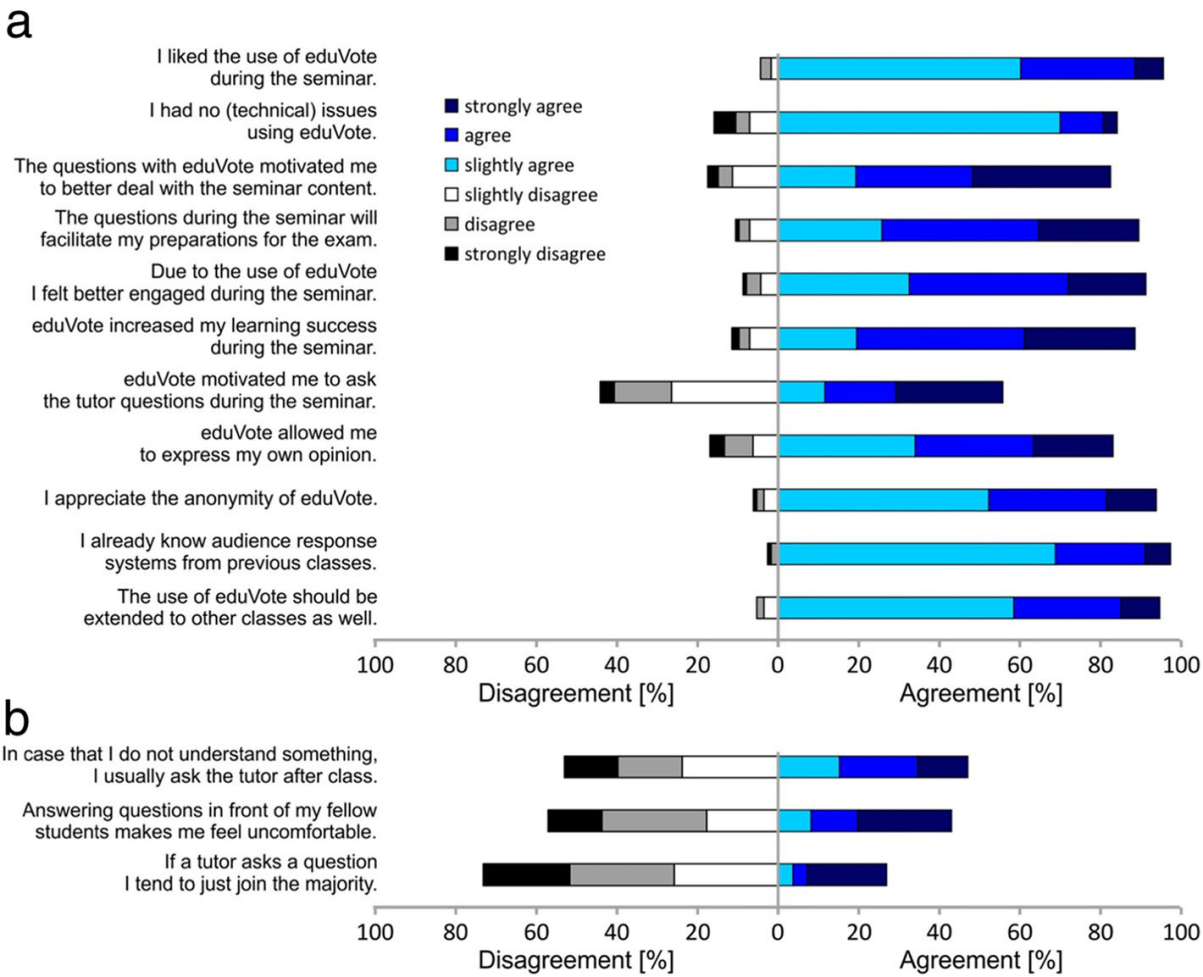
Evaluation results regarding the use of an audience response system. **a** Immediately after the respective seminar, students were asked about their agreement or disagreement to specific statements regarding the use of the audience response system eduVote using a 6-point Likert scale [12] (from 1 “strongly agree” to 6 “strongly disgree”). Shown is the agreement or disagreement to each statement in % of the answers given (*n* = 114). Overall, we noticed a high degree of satisfaction with the use of the audience response system. **b** Assessment of the students’ general perception and handling regarding questions asked by a tutor or to be addressed to a tutor. Shown is the % of agreement or disagreement to each statement

We then evaluated the use of the audience response system using anonymous questionnaires. The student’s opinion was immediately assessed after the respective seminar using a 6-point Likert scale [12] (from 1 “strongly agree” to 6 “strongly disagree”; Fig. 2, Supplementary Table 1). Most students already knew audience response systems like eduVote from previous classes (mean of 1.5 on a 6-point Likert scale ± SEM of 0.1). The students liked the use of eduVote (1.6 ± 0.1) and felt better engaged during the seminar (2.1 ± 0.1). They noticed that eduVote motivated them to better deal with the seminar content (2.6 ± 0.1), that it increased their learning success (2.4 ± 0.1) and that the questions with eduVote will facilitate their preparations for the exam (2.2 ± 0.1). They further appreciated that eduVote allowed them to express their own opinion (2.3 ± 0.1) and also appreciated the anonymity of eduVote (1.8 ± 0.1).

While we observed no major differences between young and older students, two questions revealed differences between male and female students: While only 7% of male students (1.45 ± 0.1 on a 6-point Likert scale) reported technical difficulties when using eduVote, 24% of female students had technical difficulties (2.11 ± 0.15) (*p* = 0.017). Interestingly, while 36% (4.07 ± 0.1) of male students reported to typically join the majority when a teacher asks a question, only 15% of female students (4.62 ± 0.15) do so (*p* = 0.019).

**Fig. 3:**
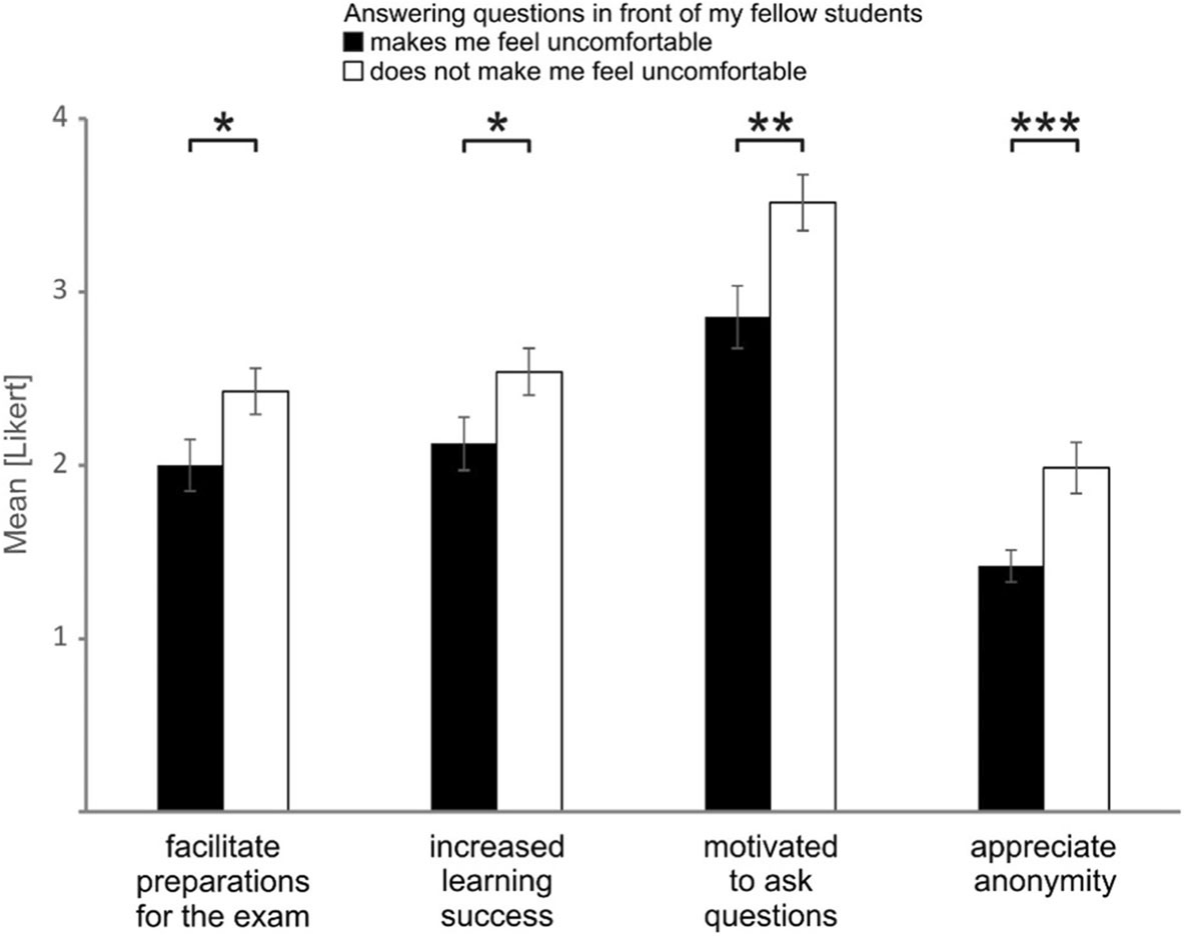
Students who feel uncomfortable with answering questions in front of others especially profited from the use of the audience response system. Students who indicated that they feel uncomfortable when answering questions in front of their fellow students especially profited from the use of the audience response system. Compared are the levels of agreement (mean Likert scale) of those students who indicated that “Answering questions in front of my fellow students makes me feel uncomfortable” (Likert scale 1–3, black bars, *n* = 48) with the answers of those students who did not agree to this statement (Likert scale 4–6, white bars, *n* = 64). Students who feel uncomfortable with answering questions in front of others showed a higher level of agreement with the statements “The questions during the seminar will facilitate my preparations for the exam.” (facilitate preparations for the exam, *p* = 0.034), “eduVote increased my learning success during the seminar.” (increased learning success, *p* = 0.044), “eduVote motivated me to ask the tutor questions during the seminar.” (motivated to ask questions, *p* = 0.008), and “I appreciate the anonymity of eduVote.” (appreciate anonymity, *p* = 0.002). *, *p* < 0.05; **, *p* < 0.01; ***, *p* < 0.005

We then asked the question whether other specific groups of students especially profit from the use of eduVote. We therefore grouped the students by their answers in the questionnaire and tested for specific differences between those groups. We identified that students who feel uncomfortable with answering questions in front of others especially profit from the use of the audience response system. We noticed for these students a higher level of agreement (2.00 ± 0.15 on a 6-point Likert scale) with the statement that the questions during the seminar will facilitate their preparations for the exam than for other students (2.43 ± 0.13). They further more agreed that eduVote increased their learning success (2.12 ± 0.15 vs. 2.54 ± 0.13) and felt more motivated by eduVote to ask the tutor questions during the seminar (2.85 ± 0.18 vs. 3.51 ± 0.16). As one may expect, students who feel uncomfortable with answering questions in front of others especially appreciated the anonymity of eduVote (1.42 ± 0.09 vs. 1.95 ± 0.15) (Fig. 3).

Most students (56%) reported that eduVote would motivate them to ask the tutor questions during the seminar. While we could not track, who asked specific questions, we recorded the total number of questions asked by the students in each seminar. We wanted to know whether the students indeed asked more questions during the seminars when eduVote was used. Surprisingly the opposite was true: We observed a tendency towards more questions asked by the students in case that no ARS was used (Fig. 1c).

Taken together, while we could not confirm that the use of the audience response system has an impact on the assessment results of students in our controlled crossover study we observed a high level of satisfaction with the use of eduVote and identified that those students hesitating to answer questions especially profit from the use of an anonymous audience response system like eduVote.

## Discussion

Our memory is aimed at making decisions and is prepared to forget [13]. Furthermore, without repetition, just 60% of newly learned material can be recalled after 20 min and after 1 h more than half of it is lost [14]. Only through repetitions and/or processing information is transferred from short term to long term memory [15]. Additionally, students attend many lectures passively without reaching an active or even interactive state of learning [16]. However, active learning has a high impact on the learning success and performance in assessments [17]. Beyond that, the attention of learners strongly declines after 20–25 min of classical teacher-centred lectures [18] requiring a change, like questions using an audience response system, in order to again raise the attention.

Moreover, audience response systems serve another important purpose: The questions using the audience response system and the following discussion of the wrong and correct answers provide the students important and prompt formative feedback during the learning process and about their learning progress [19–21] which especially enhances the positive effect of multiple-choice testing [22]. It is difficult to accurately measure the effectiveness of feedback [23]. However, one can at least state that feedback has overall a medium-high effect on student learning and is especially effective for cognitive outcome measures [24] as it activates both fast and slow learning and memory processes in the brain [25].

For these reasons, we integrated an audience response system into our seminar as audience response systems aim at knowledge required for decision making, repeat the contents taught, establish interactivity, activate students, and provide individualized formative feedback.

It was therefore fair to assume that audience response systems should also impact the results of assessments. Previous studies on the impact of audience response systems on assessments showed contradictory results [6, 7, 9]. One needs to consider that it is difficult if not impossible to form appropriate control groups if the impact of audience response systems is assessed in plenary lectures.

Here, we conducted a controlled educational research study in a seminar setting which allowed a direct comparison between topics taught with an audience response system and topics without this additional interactive part. Furthermore, the dummy coding procedure allowed us to reach a considerable high number of study participants compared with other studies [5–7, 9].

Students answered interactive questions during their classes using the audience response system eduVote. Control groups were instructed without this interactive part. We analysed the results of the final assessment of students for an impact of the use of the audience response system. However, we could not demonstrate a positive long-term impact of the audience response system on learning and perception.

It is possible that our seminar “Human Genetics” was already interactive enough and that the additional activation using the audience response system had no further effect as the participants already have reached an interactive state of learning [16]. One major bias may have been an overlearning of students in preparation to a summative assessment [26]. This effect may have covered the effect of the teaching methods on the results of the summative assessment.

Due to the nature of this educational research studies, students could not be blinded to the fact that they were exposed to the audience response system. However, as different groups were exposed to the audience response system for different topics, students were blinded when they served as control group. For organizational reasons, we could not randomly assign individual students to study arms as the groups were precomposed. However, we randomly assigned each group to the study arms. In order to exclude bias by different teachers, tutors were randomly assigned to the different seminar and control groups. In order to exclude any dropouts, we carefully tracked whether students participated at the right time, in the right group and the right room.

Strengths of our study were e.g. the large samples size with clear intervention and control cohorts consisting of multiple independent groups. Importantly, our study was not conducted in a laboratory setting with e.g. pre-recorded lectures or artificial questions but during regular classes with real students and tutors. The tutors were highly qualified and experienced. Our results were unaffected by incentives and we still noted a high motivation of the students to participate in our study and to share their opinion and exam results with us. Furthermore, eduVote turned out to be an easy-to-use and reliable audience response system and our intervention could be easily adapted into other classes and courses.

After the class, we evaluated the use of eduVote. In our questionnaire, the students gave a very positive feedback regarding the use of the audience response system. The students further stressed that they especially appreciated the anonymity of the audience response system and that they felt not to be forced to join the majority.

Interestingly, we observed that we could specifically reach and activate students who feel uncomfortable with answering questions in front of others by changing the traditional way of asking questions through the use of an audience response system. While others proposed such an effect of audience response systems before [27], we here provide evidence for this assumption.

Our results are in line with previous results indicating that the main advantage of audience response systems would be more the motivation of students and the generation of a stimulating learning environment than the improvement of assessment grades [6].

## Conclusion

Audience response systems are important tools to activate and engage students during classes. However, in our controlled education research study we could not demonstrate a long-term effect of the audience response system on assessment results. Interestingly, we observed that the audience response system specifically reaches and activates students who feel uncomfortable with answering questions in front of others which may not be reachable without such an anonymous tool.

## Supplementary information

**Additional file 1: Supplementary Table 1.** Detailed evaluation results regarding the use of eduVote. Students evaluated the use of the audience response system eduVote using a 6-point Likert scale (from 1 “strongly agree” to 6 “strongly disgree”). Shown are details for individual items/questions: The most frequent answer, the median, the mean and the standard error of means (SEM) are listed (*n* = 114).

## Data Availability

The datasets used and/or analysed during the current study are available from the corresponding author on reasonable request.

## Abbreviations

ARS: Audience response system
