# Supplementary material for: The impact of an audience response system on a summative assessment, a controlled field study
Source: BMC Med Educ. 2020 Jul 13;20:218. doi: 10.1186/s12909-020-02130-4 (PMC7359272; doi:10.1186/s12909-020-02130-4)
Supplement: Supplementary file 1 — Additional file 1: Supplementary Table 1. Detailed evaluation results regarding the use of eduVote. Students evaluated the use of the audience response system eduVote using a 6-point Likert scale (from 1 “strongly agree” to 6 “strongly disgree”). Shown are details for individual items/questions: The most frequent answer, the median, the mean and the standard error of means (SEM) are listed (n = 114). [file 12909_2020_2130_MOESM1_ESM.docx]

| **Item** | **most frequent answer** | **median** | **mean** | **SEM** |
| --- | --- | --- | --- | --- |
| I liked the use of eduVote during the seminar. | 1 | 1 | 1.6 | 0.1 |
| I had no (technical) issues using eduVote. | 1 | 1 | 1.8 | 0.1 |
| The questions with eduVote motivated me to better deal with the seminar content. | 3 | 3 | 2.6 | 0.1 |
| The questions during the seminar will facilitate my preparations for the exam. | 2 | 2 | 2.2 | 0.1 |
| Due to the use of eduVote I felt better engaged during the seminar. | 2 | 2 | 2.1 | 0.1 |
| eduVote increased my learning success during the seminar. | 2 | 2 | 2.4 | 0.1 |
| eduVote motivated me to ask the tutor questions during the seminar. | 4 | 3 | 3.2 | 0.1 |
| eduVote allowed me to express my own opinion. | 1 | 2 | 2.3 | 0.1 |
| I appreciate the anonymity of eduVote. | 1 | 1 | 1.8 | 0.1 |
| I already know audience response systems from previous classes. | 1 | 1 | 1.5 | 0.1 |
| The use of eduVote should be extended to other classes as well. | 1 | 1 | 1.6 | 0.1 |
| In case that I do not understand something,  I usually ask the tutor after class. | 4 | 4 | 3.5 | 0.2 |
| Answering questions in front of my fellow students makes me feel uncomfortable. | 5 | 4 | 3.8 | 0.1 |
| If a tutor asks a question I tend to just join the majority. | 5 | 4 | 4.3 | 0.1 |
